# Drought and Nitrogen Application Modulate the Morphological and Physiological Responses of *Dalbergia odorifera* to Different Niche Neighbors

**DOI:** 10.3389/fpls.2021.664122

**Published:** 2021-07-02

**Authors:** Li-Shan Xiang, Ling-Feng Miao, Fan Yang

**Affiliations:** ^1^School of Ecological and Environmental Sciences, Hainan University, Haikou, China; ^2^School of Forestry, Hainan University, Haikou, China; ^3^Center for Eco-Environmental Restoration Engineering of Hainan Province, Haikou, China; ^4^Key Laboratory of Agro-Forestry Environmental Processes and Ecological Regulation of Hainan Province, Haikou, China

**Keywords:** niche difference, biomass accumulation and allocation, root system interaction models, morphological and physiological response, root system isolated model

## Abstract

Mixed stands can be more productive if growth facilitation via niche segregation occurs. *Dalbergia odorifera* T. Chen, a tropical tree species endemic to Hainan Island with great economic values, belongs to the family Leguminosae. However, selecting mixed species with suitable ecological niches to efficiently construct mixed forests of *D. odorifera* in the context of abiotic stress [drought, nitrogen (N) deposition] remained obscure. In the present study, the target plant *D. odorifera* was planted with the same species *D. odorifera*, heterogeneous but the same family *Delonix regia* and non-Leguminous Family *Swietenia mahagoni* in the root interaction and isolated models under two watering regimes [100% and 30% field capacity (FC)] and two N applications (application, non-application), respectively. Principle component analysis based on the performances of growth, phenotype, and physiology was performed to identify the main factors affected by the treatments and the most discriminatory effects of water, N level, and species interaction models. Both comprehensive evaluation values and comprehensive index values were calculated to evaluate the influences of different niche neighbors on *D. odorifera*. Results showed that *D. odorifera* was benefited from *S. mahagoni* but inhibited from *D. odorifera* in all treatments under root system interaction. Drought stress aggravated the inhibitory effects on *D. odorifera* from *D. odorifera*. N application stimulated the promoted effects on *D. odorifera* from *S. mahagoni* but enhanced competition intensity of *D. odorifera* from *D. regia* under the 100% FC condition. N application alleviated the inhibitory effect of drought stress on *D. odorifera* from *D. odorifera* and *S. mahagoni*. Furthermore, the responses of *D. odorifera* to different niche neighbors were dominated by belowground interaction rather than the negligible aboveground one. Therefore, the feasibility of niche segregation as the criterion for selecting neighbors to construct *D. odorifera* mixed stands was confirmed. In addition, water level and N application could alter responses of *D. odorifera* to different niche neighbors under the root system interaction. Appropriate N application could alleviate the inhibitory effect of drought stress on *D. odorifera* in its mixed forests. A mixture with *S*. *mahagoni* under appropriate N application could be the optimal planting model.

## Highlights

- *Dalbergia odorifera* benefitted from *Swietenia mahagoni* but was inhibited by the conspecifics.
- Drought aggravated the inhibitory effects on *D. odorifera* from the conspecifics.
- Nitrogen (N) application stimulated the positive effects on *D. odorifera* from *S. mahagoni* but enhanced competition intensity of *D. odorifera* from *Delonix regia* under well-watered conditions.
- N application alleviated the inhibitory effects of the drought stress on *D. odorifera* from the conspecifics and *S. mahagoni*.
- Negligible aboveground competition occurred in root isolated models.

## Introduction

Interaction among species affects plant growth and community structure (Goisser et al., 2016; Guo et al., 2016). Compared with the traditional pure stands, mixed stands had greater ecological advantages such as higher productivity, biodiversity, and resistance to abiotic stresses in forest management (Richards et al., 2010; Nguyen et al., 2015; Forrester and Bauhus, 2016; Liu et al., 2018). The reduction of competition or facilitation has been proposed to explain the increased productivity from mixed stands (Chomel et al., 2014; Forrester and Bauhus, 2016). Facilitation refers to the fact that a species promotes the growth of its neighboring species by improving the environment [e.g., nitrogen (N) fixation from leguminous species]. Niche segregation is often the basis to select species to reduce competition when constructing mixed stands, such as inter-specific differences in functional traits, resource demand, and absorptive capacity (e.g., shade tolerance, growth rate, root morphology, root structure, nitrogen fixation capacity, nutrient preference) (Forrester and Bauhus, 2016). Also, niche complementarity theory implies that greater niche separation among neighbors may produce more favorable benefits during interaction (File et al., 2012). For example, the interaction among the same species and one among different species belonging to the same genus have different effects on the growth because of different niches in resource utilization (Burns and Strauss, 2011; Bowsher et al., 2017). Thus, niche separation in structural and functional traits via competitive reduction is generally regarded as the primary criteria in selecting neighboring species for mixed forests, and it is widely used to enhance plant growth, productivity, and adaptability to abiotic stresses during plantation forest management (Richards et al., 2010; Pretzsch et al., 2013; Nguyen et al., 2015; Liu et al., 2018).

There is considerable variability during the processes of species interaction in mixed stands along with resource availability or climatic conditions (Forrester, 2014; Goisser et al., 2016; Svanfeldt et al., 2017; Calama et al., 2019). Water deficiency and N deposition are very common with the aggravation of global climate change (Goisser et al., 2016; Liang et al., 2019). It exerted strong impacts on forest ecosystems, individual tree growth, and community composition by species competition (Yi et al., 2015; Aldea et al., 2017; Calama et al., 2019; Helluy et al., 2020). Related studies have confirmed that the interactive relationship among neighboring species can be modified by the variations of abiotic factors, such as belowground resource competition for soil water and nutrient and aboveground competition for light source (Craine et al., 2013; Pierik et al., 2013). For example, the declined growth and death of fir are affected by the increasing competition resulted from abiotic stresses (Linares et al., 2009). Therefore, whether the method of selecting mixed-species based on niche separation will continue to work despite various resource levels (water and N) must be considered.

Water is a key resource that determines individual growth, stand productivity, and dynamics of competition. The low water availability can induce belowground competition for water resources. Related studies have confirmed the adaptive strategy of neighboring species and the outcome of competition would be modified by drought stress in mixed plantations (Wright et al., 2015; Goisser et al., 2016; Aldea et al., 2017; Helluy et al., 2020). For example, in response to drought stress, the plant may maximize root length and root surface area and modify root depth or placement to access more water (Aschehoug et al., 2016); The responses from the same species under a water-limited condition were aggravated because of the similarity of resource utilization (Chen et al., 2014; Calama et al., 2019). The competitive effect of neighbors of *Arrhenatherum elatius* was transformed into a facilitative effect when the plants were exposed to drought (Grant et al., 2014).

Abiotic factors such as water, nutrition, and light may occur simultaneously and interact strongly (Niinemets, 2010). It implies that the species interaction for the combined nutrients and light or the combined nutrients and water has a greater regulatory effect on plant behavior than the single competition (Mittler, 2006; Niinemets, 2010; Forrester, 2014). Light competition causes the increment in aboveground biomass and the reduction in belowground investment, possibly because light requirement affects belowground tissue competition for water and nutrients (Aschehoug et al., 2016). For example, the field experiment on the *Agropyron desertorum* shows that light affects the belowground root structures and resource distribution when plants are exposed to compound stresses of both aboveground and belowground competition (Bilbrough and Caldwell, 1995). Accordingly, transformation, synergy, or interaction relationships may be found among aboveground and belowground competition processes. Our study investigated whether the aboveground interaction (e.g., light competition) can affect growth and underground competition via root system isolated experiments.

The previous studies have reported that Leguminous plants have different responses to N addition. Some Leguminous species could keep homeostasis after N addition due to N acquisition via both absorption from the soil and biological N fixation (Markham and Zekveld, 2007; Guo et al., 2017; Wang et al., 2018). However, other Leguminous species are sensitive to N deposition (Hansen et al., 1992) and converted the symbiont N fixation strategy into soil N absorption with sufficient N application (Markham and Zekveld, 2007; Wang et al., 2019). In addition, there are substantial pieces of evidence that N application can modify the N transfer and the interaction in mixed stands, such as non-leguminous *Eucalyptus urophylla* × *Eucalyptus grandis* and leguminous *Dalbergia odorifera* T. Chen (Yao et al., 2019). The related study indicated that Leguminous species play a key role in measuring the impacts of various abiotic environments on the dynamics of forestry systems (Wang et al., 2018). Therefore, under the background of precipitation reduction and N deposition increase, the effects of simulated N deposition increase on the growth and development of Leguminosae Family *D. odorifera* in mixed forests should be further studied.

*D. odorifera*, a tropical tree species endemic to Hainan Island that has great economic values (Chan et al., 1998; Yu et al., 2007), belongs to the family Leguminosae. At present, *D. odorifera* is widely popularized and cultivated in the subtropical regions in China (Meng et al., 2010; Sun et al., 2015). However, the pure forest of *D*. *odorifera* does not grow well in these regions because of drought and the lack of suitable neighbors. Therefore, it should be encouraged that an optimal mixed-plantation model of improving growth and development of *D*. *odorifera* and stimulating N fixation under drought and N deposition. *Delonix regia* and *Swietenia mahagoni* are also tropical woody plants with medical and economic values (Rodan et al., 1992; Guevara et al., 1996; Mishra et al., 2011), which belong to the Leguminosae Family and different family of *D*. *odorifera*, respectively. Accordingly, the degree of niche differentiation among these three tree species gradually increases with the variation trend of plants from species to family. Collectively, constructing the *D*. *odorifera* plantation and exploring its optimal plantation models via the combination of abiotic factors (water and N) and species interaction is important.

In the present study, we hypothesized that (1) *D. odorifera* would differentially respond to different niche neighbors under the root system interaction or isolation conditions, (2) the responses of *D. odorifera* to different neighbors would be modulated by the N application and drought stress or combined application, and (3) *D. odorifera* would be influenced by the belowground interaction from different neighbors rather than aboveground interaction.

## Materials and Methods

### Plant Materials and Experimental Designs

In the present study, *D. odorifera* of Family Leguminosae was used as target species, *D. regia* and *S. mahagoni* were used as different related-niche neighbors of *D. odorifera*. Moreover, responses to different niche neighbors and interactions between aboveground and belowground tissues under water deficiency and N application were considered. The whole experiments were conducted in a greenhouse at the Hainan University, Haikou City, China (20° 03′ 33.2″ N, 110° 20′ 16.9″ E), where the climate is characterized by a mean annual temperature, mean annual precipitation, and relative humidity are 24.3°C, 1,684 mm, and 85%, respectively.

A total of 720 1-year-old seedlings (480 *D. odorifera* seedlings, 120 *D. regia* seedlings, and 120 *S. mahagoni* seedlings) were collected from the local nursery garden in Jianfengling (18° 42′ 57.91″ N, 108° 52′ 18.65″ E), Ledong County, Hainan Province, China. All selected healthy seedlings were kept approximately the same basal stem diameter and height (20 cm) to reduce asymmetries in competition caused by differences in plant size. The climatic conditions of Jianfengling are similar to the experimental site.

**Figure 1:**
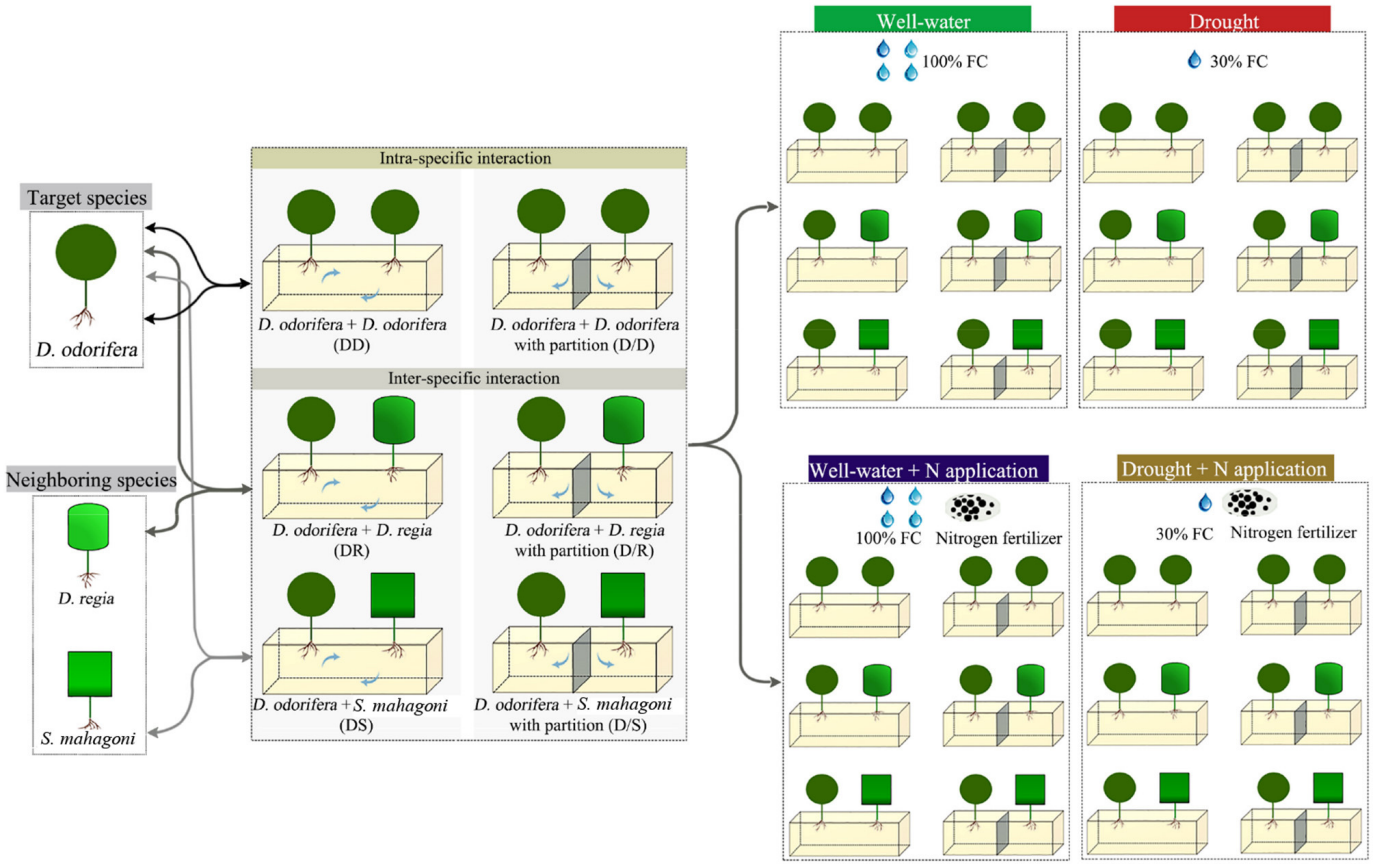
Schematic diagram of the experimental design.

The experimental layout was completely randomized with three factors (species interaction, water regimes, and N fertilizer application), as shown in Figure 1. One *D. odorifera* seedling and one neighboring species seedling were transplanted in a plastic pot (50 × 21 × 16 cm, length × wide × height) at the end of February 2018. Plastic pots filled with 15 kg homogenized soil (red soil: sand = 1:2, v/v). The distance between pots was maintained at 100 cm to ensure that they are not affected by each other. Two individuals (two *D. odorifera* seedlings, one *D. odorifera* seedling and one *D. regia* seedling, or one *D. odorifera* seedling and *S. mahagoni* seedling) per pot were spaced ~25 cm apart. In order to eliminate the damages to the original root system of seedlings during transplanting, each seedling was transferred with the container soil (about 80 g dry weight) together after removing the plastic bag.

Two kinds of pots were used for the experiment to provide two types of root interaction and isolation: pots were separated into two isolate parts by a plastic partition in the middle of the pot to exclude root interaction between *D. odorifera* and neighboring species, i.e., only aboveground interaction was allowed between them; the other pot was without partition and so belowground root interaction was possible, i.e., where the two species could interact from aboveground parts and belowground roots simultaneously. So, species interaction included three treatments under belowground root system interaction models with no-partition pots (one intra-specific interaction, *D. odorifera* + *D. odorifera*, DD; one inter-specific interaction between species belonging to different genus but the same family, *D. odorifera* + *D. regia*, DR; one inter-specific interaction between species belonging to a different family, *D. odorifera* + *S. mahagoni*, DS). Accordingly, another three treatments underground root system isolated models with partition pots (*D. odorifera* + *D. odorifera*, D/D; *D. odorifera* + *D. regia*, D/R; *D. odorifera* + *S. mahagoni*, D/S) were set up.

A full-strength Hoagland's solution was supplemented regularly to ensure the nutrient requirement during the cultivation of *D. odorifera*. After growing steadily for 4 weeks, two watering regimes [well-watered condition, 95–100% field capacity (FC); drought-stressed condition, 25–30% FC] and two N applications (application and non-application) treatments were carried out. N fertilizer was provided as NH_4_NO_3_ and was dissolved in pure water (the concentration of N solution was 0.64 and 3.2 g/L). The N treatments included no-N application and N application. In the N application treatment, 1,000 mL 0.64 g/L N solution in the well-watered condition and 200 mL 3.2 g/L N solution in the drought condition were poured into each pot, respectively. Accordingly, N solution was replaced by equal volume pure water in the no-N application treatment. N solution was irrigated to the pots once a week during the experiment. The pots were weighed every day to keep 95–100% FC in the well-watered treatments and 25–30% FC in the drought treatments. A total of 24 treatments were performed. Five replications, three pots in each replication, were included in each treatment. After 120 days of treatment, the plants were harvested (Supplementary Figure 1).

### Determination of Growth and Phenotypic Traits

At the end of the experiment, five pots from each treatment were selected randomly to measure the height (H), stem diameter (D), the number of branches and total leaves, and leaf area (LA). The LA was measured by an LI-3000 area meter (LI-3000C, LI-COR, USA). Then, the selected *D. odorifera* were harvested and divided into leaves, stems, and roots. Biomass samples were dried to a constant weight at 70°C. The leaf dry matters (LDM), shoot dry matters (SDM), and root dry matters (RDM) were then determined. The total dry matter (TDM) was the sum of the LDM, SDM, and RDM. The ratio of leaf to stem dry matters (L/S) was calculated as LDM divided by the SDM. The ratio of leaf to root dry matters (L/R) was calculated as LDM divided by the RDM. The ratio of stem to root dry matters (S/R) was calculated as SDM divided by the RDM. The ratio of belowground to aboveground dry matters (B/A) was calculated as belowground tissue dry matters divided by the aboveground tissue dry matters (the sum of the leaf and stem dry matters); the specific leaf weight (SLW) was calculated as the ratio of leaf weight to LA.

### Gas Exchange Measurements

The fourth fully expanded leaf of *D. odorifera* from each replication was used to measure the gas exchange with the LI-6400 portable photosynthesis measuring system (LI-6400XT, Gene Company, USA) according to the methods of Xu et al. (2008). The net photosynthetic rate (*Pn*), stomatal conductance (*Gs*), intercellular CO_2_ concentration (*Ci*), and transpiration rate (*Tr*) were measured in controlled conditions between 08:00 and 11:00. The red and blue light source was used, and light source intensity was set to 1,200 μmol·m^−2^·s^−1^. The intrinsic water-use efficiency (WUEi) was calculated as the ratio of *Pn* to *Tr*. N concentration in the leaves was determined by the semi-micro Kjeldahl method (Mitchell, 1998). Afterward, photosynthetic N-use efficiency (PNUE) was further calculated as *Pn* per leaf N content per area.

### Determination of Activities of Catalase (CAT), Superoxide Dismutase (SOD), and Peroxidase (POD), and Proline Content

The fresh leaf samples from five randomly chosen individuals in each treatment were collected for CAT, SOD, and POD analyses at the end of the experiment. The CAT, SOD, and POD activity were measured as described by Yang et al. (2011). A sample of 0.2 g leaves was grounded with liquid N and homogenized in 10 mL of 100 m mol/L universal sodium phosphate extraction buffer. Details of the extraction buffer have been described by Han et al. (2015). After centrifugation, the supernatant was used to determine proline, as Yang et al. (2015) described.

### Determination of Photosynthetic Pigment Contents, Relative Water Content, and Leaf Non-Structural Carbohydrate Content

About 0.2 g fresh leaves from each replication were used to determine chlorophyll a, chlorophyll b, and carotenoids contents. These fresh leaves were extracted in 95% (v/v) ethanol. The absorbance values were measured with the spectrophotometer (UV-1800PC, Shanghai Meipda Instrument Co., Ltd, China) at 470, 649, and 665 nm after being placed in the dark box for 24 h. The absorbance values were converted to chlorophyll and carotenoid concentrations described by Zhang et al. (2016a,b). The RWC of the leaves, stems, and roots were calculated by fresh weight and dry weight according to the method of González and González-Vilar (2001). The dried leaves were ground to powder to determine soluble sugar and starch content according to the procedure of the anthrone-sulfuric acid method (Yemm and Willis, 1954) and the anthrone reagent (Yemm and Willis, 1954) using glucose as the standard, respectively. About 50 mg of dry powdered plant samples in 10-mL centrifuge tubes were mixed with 5 mL of 80% (v/v) ethanol, incubated in a water bath at 80°C for 30 min, and centrifuged at 5,000 rpm for 15 min. This procedure was repeated twice, and the supernatants pooled together for the total soluble sugar content measurement. The solid residues left in the centrifuge tubes after soluble sugar extraction were dried in a vacuum drier at 80°C for 24 h to the determination of starch. The reaction in the tubes was accelerated by heating in a boiling water bath for 15 min after adding 4 mL of hot distilled water. Then, the sample was hydrolyzed with 4 mL perchloric acid for 15 min after cooling, followed by centrifugation at 2,500 rpm for 15 min. Samples were then extracted twice with perchloric acid, and the supernatants were combined for starch content measurement by the anthrone reagent. NSC was calculated by the sum of total starch contents and soluble sugar contents.

### Data Analysis

The competition index could assess intra-specific and inter-specific relations among three tested species, which could be quantified by relative competition intensity (RCI). The RCI on *D. odorifera* was calculated by the following equation (Jolliffe, 2000):

$$\begin{array}{l} \text{RC}{\text{I}}_{\text{ab}}=\left({\text{Y}}_{\text{ab}}-{\text{Y}}_{\text{aa}}\right)/{\text{Y}}_{\text{aa}}, \end{array}$$

Y_ab_ and Y_aa_ are the average total biomass of *D. odorifera* under inter-specific interaction and total biomass under intra-specific interaction. The growth of *D. odorifera* was considered to be promoted and subjected to positive effects by interaction from neighbors when RCI_ab_ > 0 but led to diametrically opposite results when RCI_ab_ < 0. In addition, the high value of RCI_ab_ indicated the strong positive effect of inter-specific interaction on the growth of *D. odorifera*.

Principal component analysis (PCA) was used to identify the main factors affecting *D. odorifera* by treatments (water, N application, and species interaction models) and the most discriminatory effects of these treatments. PCA based on trait combinations (growth, phenotypic and physiological indexes (height, stem diameter, the numbers of branches, total leaves, etc.) and Pearson correlation coefficient were conducted using the canoco5.0 (Microcomputer Power, Ithaca, NY). The results were expressed as mean ± standard error. Mean values (height, stem diameter, the numbers of branches, and total leaves. etc.), from each replication were compared using linear mixed models. In addition, mixed models were also conducted to assess the effects of water, N application, species interaction, and their interactions on each trait. The water regimes, N application, and species interaction were used as fixed factors. Linear mixed models were used for all response variables. Tukey's test performed multiple comparison analyses. Before statistical analyses, homoscedasticity of variances and normality of distributions was checked for each variable by Levene's test and Shapiro-Wilk's test, respectively, and log-transformed were applied to correct for deviations from these assumptions when needed. All statistical effects were considered significant at *P* < 0.05. Linear mixed models were implemented using SPSS 19.0 for Windows statistical software package (SPSS, Chicago, IL).

The comprehensive evaluation value (*E*) and comprehensive index value (*C*) were used to evaluate the effects of different neighboring species on the *D. odorifera* in the same water regimes and N application under the root system interaction models. *E* and *C* were calculated based on the phenotypic and physiological traits according to the following equation (Fang et al., 2017):

(1) $$\begin{array}{r} X\left(\mu \right)=\left(\text{C}\left(\mu \right)-X_{\text{min}}\right)/\left(X_{\text{max}}-X_{\text{min}}\right), \end{array}$$

(2) $$\begin{array}{r} \bar{X_{i}}=\frac{1}{\text{n}}\sum_{\text{j}=1}^{n}\;X_{ji}, \end{array}$$

(3) $$\begin{array}{c} I_{i}=\sqrt{{\sum^{}}_{j=1}^{n}{\left(X_{ji}\;-\;\bar{X_{i}}\right)}^{2}}/\bar{X_{i}}, \end{array}$$

(4) $$\begin{array}{r} W_{i}=I_{i}/\sum_{\text{i}=1}^{m}I_{i}, \end{array}$$

(5) $$\begin{array}{r} E=\sum_{\text{i}=1}^{n}\left[X\left(\mu \right)\;\times \;W_{i}\right], \end{array}$$

where *X*(μ), C(μ), *X*_min_, and *X*_max_ in formula (1) are the subordinate function value, observed value, and the minimum and maximum value of the μth comprehensive indicator, respectively. *X*_*i*_, n, and *X*_*ji*_ in formula (2) represent the mean of the *i*th evaluation index, the number of interaction models (DD, DR, and DS), and the *i*th evaluation index of the *j*th interaction models, respectively. *I*_*i*_ and *X*_*ji*_ in formula (3) is the coefficient of the standard deviation of the *i*th evaluation index and the *i*th evaluation index of the interaction models. *W*_*i*_ and *I*_*i*_ in formula (4) are the weighted coefficient and contribution rate of the *i*th comprehensive indexes. The *E* value in formula (5) indicates the comprehensive evaluation value for response to different neighbors for *D. odorifera* under the same water and N application. A high *E* value suggested the remarkable promoted effect from a neighbor on *D. odorifera* in a given condition. *E* values were calculated according to the formulas (1–5) through SPSS 19.0.

## Result

### PCA Analysis

**Figure 2:**
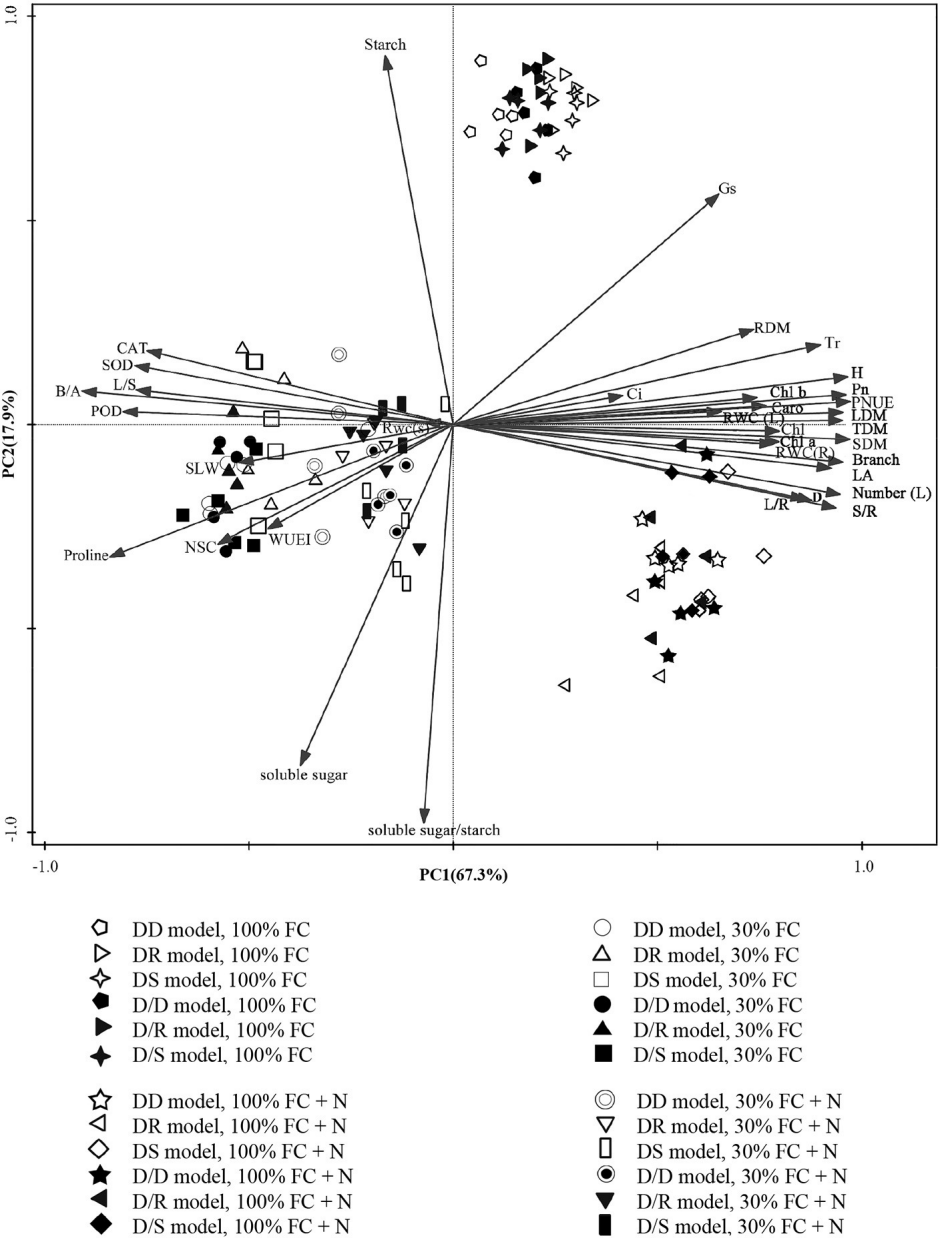
PCA of *Dalbergia odorifera* in different water, nitrogen application, and species interaction according to growth, phenotypic and physiological properties. PC1, the first principal component; PC2, the second principal component; 100% FC, 100% field capacity; 30% FC, 30% field capacity; 100% FC + N, 100% field capacity and N application treatment; 30% FC + N, 30% field capacity and N application treatment; DD, DR, and DS indicated that *D. odorifera* planted with *D. odorifera, D. regia*, and *S. mahagoni* under the root system interaction, respectively; D/D, D/R, and D/S indicated that *D. odorifera* planted with *D. odorifera, D. regia*, and *S. mahagoni* under the root system isolation, respectively.

The PCA showed a clear description of the combined phenotypic and physiological properties of *D. odorifera* in different planting models under different water regimes and N application conditions (Figure 2). SPSS extracted the two principal components, and the accumulative variance contribution was 85.2%. *D. odorifera* seedlings under 100% FC (in the first quadrant), 100% FC + N (in the fourth quadrant), and 30% FC and 30% FC + N treatments (both treatments were in the second and third quadrants but could be separated from each other) indicated that the seedlings were affected by water and N regime conditions. Under the root system interaction models, *D. odorifera* from DR (*D. odorifera* + *D. regia*) and DS (*D. odorifera* + *S. mahagoni*) models under 100% FC and 30% FC conditions were not separated from each other, whereas *D. odorifera* from these inter-specific interaction models and the intra-specific interaction model (*D. odorifera* + *D. odorifera*, DD) could be distinguished from each other. The majority of *D. odorifera* from the DR model and DS model were separated under N application conditions. However, *D. odorifera* among the interaction models under the root system isolated models (D/D, D/R, and D/S) were hard to distinguish from each other (Figure 2), indicating that *D. odorifera* under the root system isolated models could be almost insensitive to neighbors. In addition, the PCA showed that height, leaf number, number of branches, LA, biomass accumulation and allocation, gas exchange, NSC, WUEi, antioxidant enzyme activities, and proline content were the main effects on PC1. PC2 was primarily affected by soluble sugar, starch, and the ratio of soluble sugar to starch. In addition, height, number of leaf and branches, dry matter accumulation, and PNUE showed a positive correlation with *Pn* while had negative correlations with the WUEi, NSC, B/A ratio, antioxidant enzymes activities, and proline content.

### Effects of Water, N Application, and Interaction on the Growth

**Figure 3:**
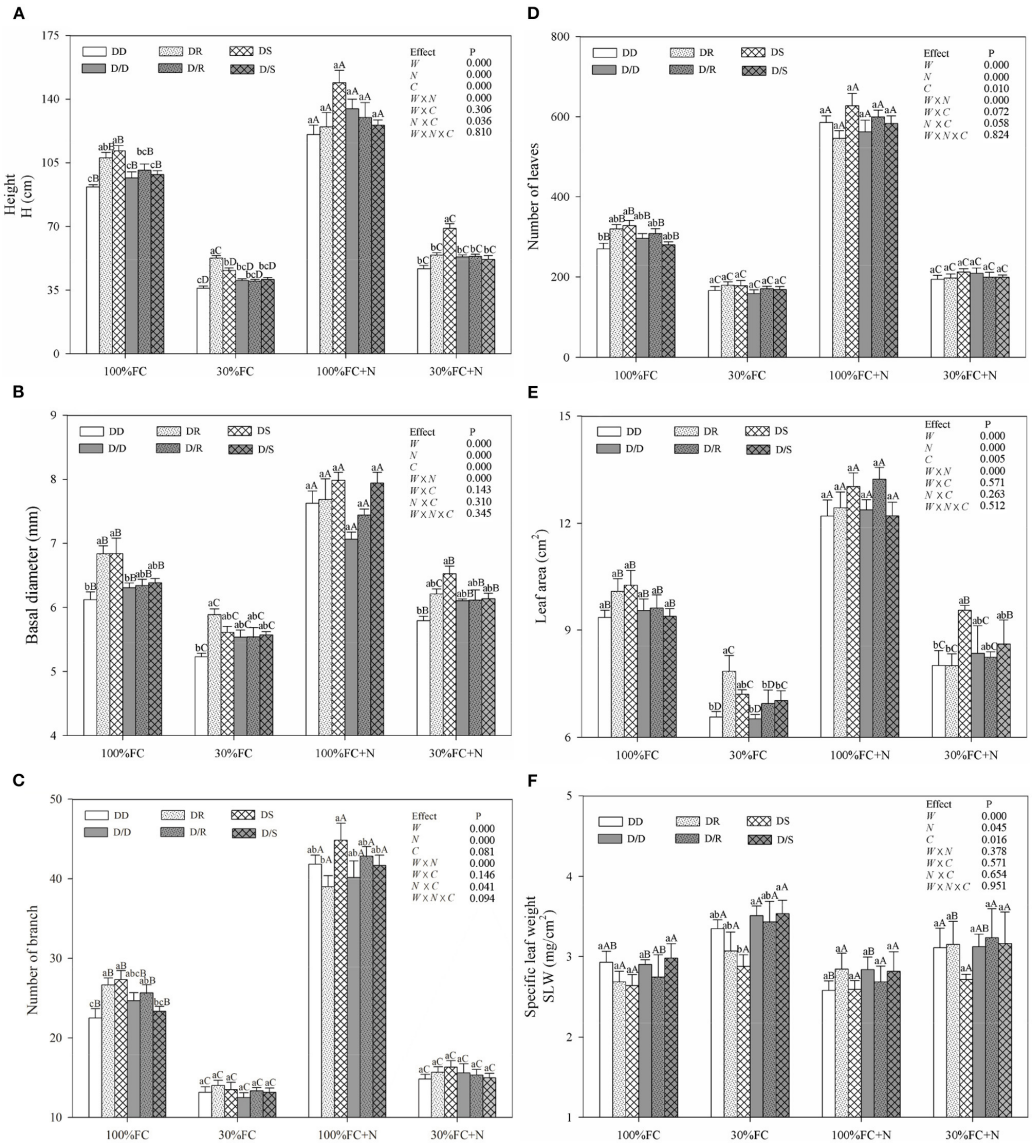
Effects of water, N application, and species interaction on height **(A)**, basal diameter **(B)**, number of branch **(C)**, leaves **(D)**, leaf area **(E)**, and specific leaf weight **(F)** of *D. odorifera*. 100% FC, 100% field capacity; 30% FC, 30% field capacity; 100% FC + N, 100% field capacity and N application treatment; 30% FC + N, 30% field capacity and N application treatment; DD, DR, and DS indicated that *D. odorifera* planted with *D. odorifera, D. regia*, and *S. mahagoni* under the root system interaction, respectively; D/D, D/R, and D/S indicated that *D. odorifera* planted with *D. odorifera, D. regia*, and *S. mahagoni* under the root system isolation, respectively; *W*, water effect; *N*, nitrogen effect; *C*, species interaction effect; *W* × *N*, the interaction effect of water and nitrogen; *W* × *C*, the interaction effect of water and species interaction; *N* × *C*, the interaction effect of nitrogen and species interaction; *W* × *N* × *C*, the interaction effect of water, nitrogen, and species interaction; Mixed model was conducted to evaluate the influence of different factors and their interaction effects. Values followed by different lowercase above the bars are significantly different at *P* < 0.05 among different species interaction models under the same water and N application; Values followed by different uppercase above the bars are significantly different at *P* < 0.05 among different water and N fertilization application under the same interaction models.

**Figure 4:**
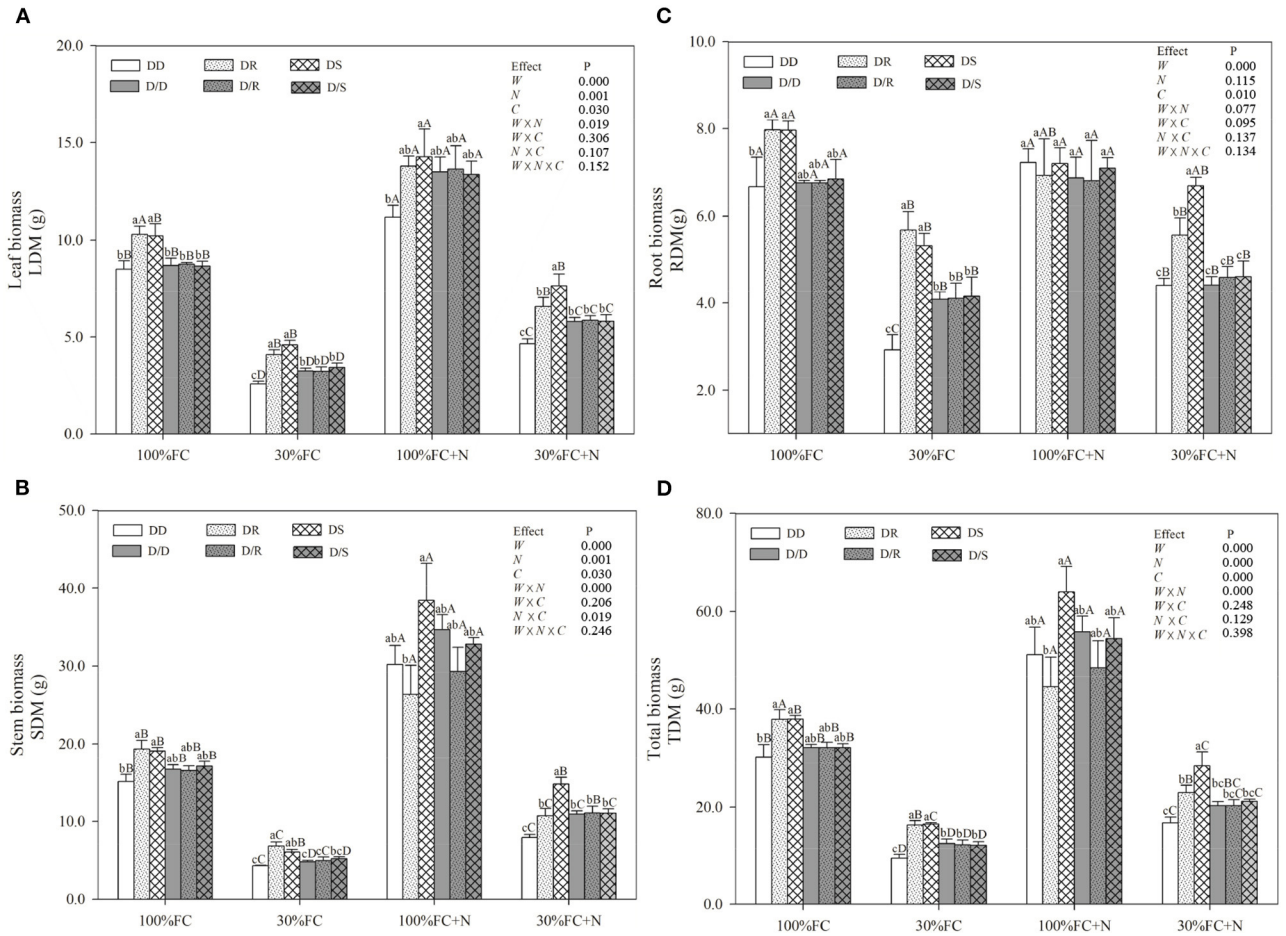
Effects of water, N application, and species interaction on leaf biomass **(A)**, stem biomass **(B)**, root biomass **(C)**, and total biomass **(D)** of *D. odorifera*. Treatments of water, N application, species interaction, data description, and statistics are shown in Figure 3.

**Figure 5:**
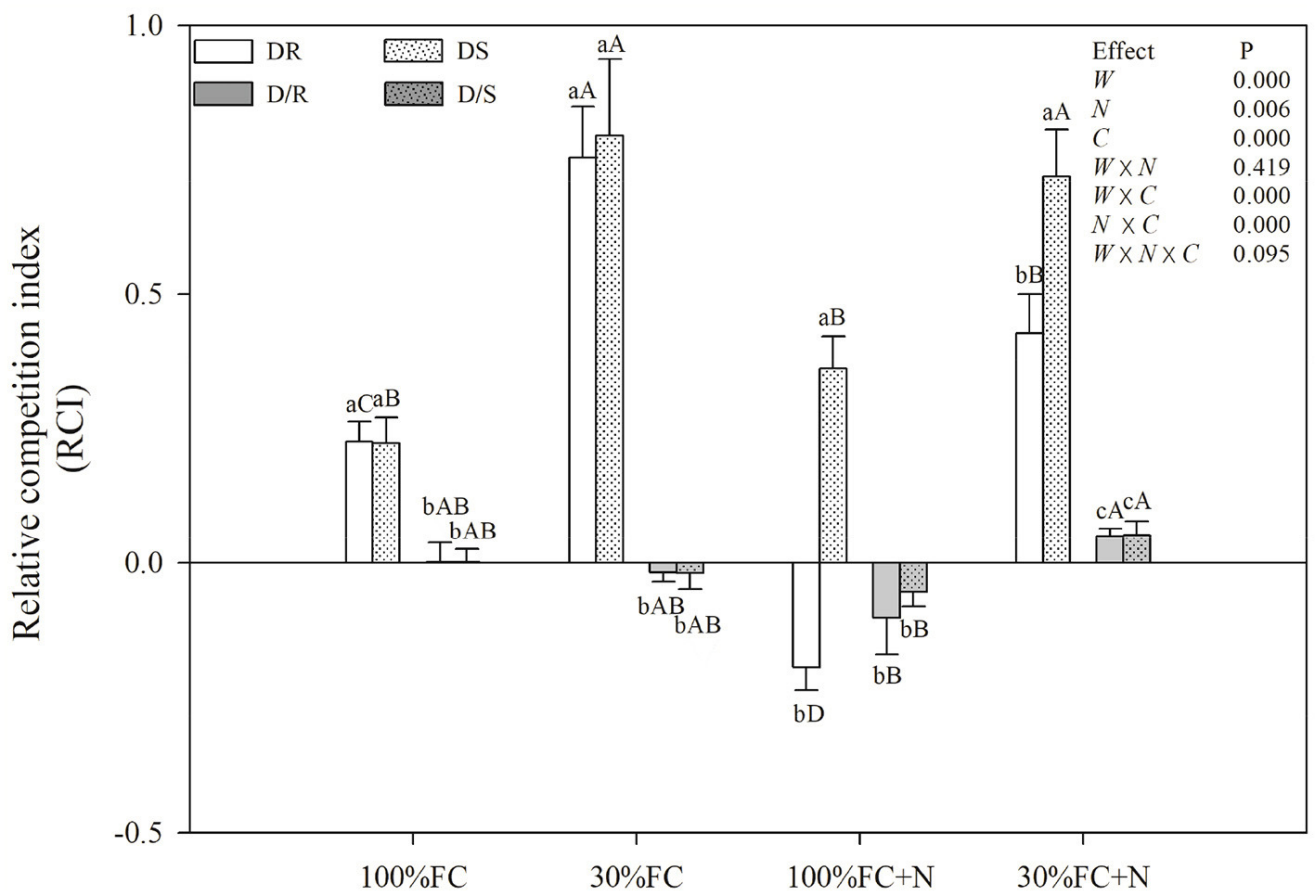
Effects of water and N application on RCI of *D. odorifera* under different species interaction models. DR and DS, *D. odorifera* interaction with *D. regia* (white bars), *S. mahagoni* (white bars with dots) under the root system interaction, respectively; D/R and D/S, *D. odorifera* interaction with *D. regia* (gray bars), *S. mahagoni* (gray bars with dots) under the root system isolation, respectively; other information about treatments, data description, and statistics are shown as in Figure 3.

Under the root system interaction models, drought-stressed *D. odorifera* seedlings showed lower H, the number of branches and total leaves, LA (Figure 3), biomass accumulation (Figure 4), and S/R (Table 1), but higher B/A and RCI values (Figure 5) compared with well-watered seedlings. In addition, greater decreases in H, LA, LDM, SDM, RDM, and TDM and less increase in B/A in drought-stressed *D. odorifera* seedlings under the DD model were found compared with those seedlings under DR and DS models (Table 2). Moreover, *D. odorifera* under the DR and DS models showed higher values in H, LA, LDM, SDM, and TDM than those under the DD model in 100% and 30% FC conditions. N application caused a significant increase in the H, the number of branches and leaves, LA, LDM, SDM, TDM, and S/R of *D. odorifera* seedlings but decreased B/A under 100% FC condition. Under the 100% FC + N conditions, *D. odorifera* seedlings under the DS model showed the highest values in H, the number of branches and leaves, LDM, SDM, TDM, and RCI in all interaction models under the root system interaction, whereas *D. odorifera* under the DR model showed the lowest SDM, TDM, and RCI (RCI <0). Under the 30% FC + N conditions, *D. odorifera* seedlings under the DD and DS models showed greater increases in the H, the number of branches, LA, LDM, SDM, RDM, and TDM than that of seedlings under the DR model (Figures 3A,E, 4A,C,D, Table 2). Moreover, H, LA, SDM, LDM, RDM, and TDM of *D. odorifera* increased as the DD, DR, and DS models under the 30% FC + N condition (Figures 3A,E, 4). Collectively, these growth and development characteristics of *D. odorifera* were significantly affected by the interaction of water, N application, and species interaction models.

**Table 1:** Biomass allocation of *D. odorifera* when exposed to different water, N application, and species interaction models.

**Water and fertilizer level**	**Species interaction model**	**L/S ratio (g/g · DW)**	**L/R ratio (g/g · DW)**	**S/R ratio (g/g · DW)**	**B/A ratio (g/g · DW)**
100% FC (W)	DD	0.58 ± 0.03Ba	1.31 ± 0.09Ba	2.28 ± 0.12Ba	0.28 ± 0.01Ca
	DR	0.53 ± 0.02Aa	1.29 ± 0.04Ba	2.46 ± 0.13 Ba	0.27 ± 0.01 Ba
	DS	0.53 ± 0.02Ba	1.26 ± 0.05Ba	2.40 ± 0.07Ba	0.27 ± 0.01Ca
	D/D	0.52 ± 0.02Ba	1.29 ± 0.05Ba	2.47 ± 0.04Ba	0.27 ± 0.01Ba
	D/R	0.53 ± 0.01Ba	1.29 ± 0.02Ba	2.44 ± 0.08Ba	0.27 ± 0.01Ba
	D/S	0.51 ± 0.02Ca	1.27 ± 0.02Ba	2.52 ± 0.10Ba	0.27 ± 0.01Ca
30% FC (D)	DD	0.63 ± 0.03Aa	0.86 ± 0.05Da	1.36 ± 0.06Da	0.46 ± 0.03Aa
	DR	0.62 ± 0.03Aa	0.76 ± 0.06Ca	1.23 ± 0.07Ca	0.51 ± 0.02Aa
	DS	0.70 ± 0.02Aa	0.88 ± 0.04Ca	1.26 ± 0.06Ca	0.47 ± 0.02Aa
	D/D	0.67 ± 0.01Aa	0.81 ± 0.01Ca	1.20 ± 0.02Ca	0.50 ± 0.02Aa
	D/R	0.65 ± 0.02Aa	0.80 ± 0.04Ca	1.24 ± 0.06Ca	0.50 ± 0.03Aa
	D/S	0.66 ± 0.02Aa	0.86 ± 0.09Ca	1.31 ± 0.12Ca	0.48 ± 0.03Aa
100% FC + N (WN)	DD	0.38 ± 0.03Cb	1.57 ± 0.06Aa	4.22 ± 0.33Aa	0.18 ± 0.01Da
	DR	0.55 ± 0.06Aa	2.23 ± 0.46Aa	3.97 ± 0.47Aa	0.17 ± 0.02Ca
	DS	0.38 ± 0.03Cb	1.78 ± 0.22Aa	4.72 ± 0.43Aa	0.16 ± 0.02Da
	D/D	0.40 ± 0.04Cb	1.97 ± 0.09Aa	4.90 ± 0.26Aa	0.15 ± 0.01Ca
	D/R	0.47 ± 0.03Bab	2.07 ± 0.17Aa	4.42 ± 0.35Aa	0.16 ± 0.01Ca
	D/S	0.41 ± 0.02Dab	1.89 ± 0.09Aa	4.63 ± 0.14Aa	0.15 ± 0.00Da
30% FC + N (DN)	DD	0.57 ± 0.02Aa	1.02 ± 0.06Cb	1.77 ± 0.08Cc	0.36 ± 0.02Ba
	DR	0.58 ± 0.03Aa	1.09 ± 0.05Bab	1.89 ± 0.08Bbc	0.34 ± 0.01Bab
	DS	0.54 ± 0.04Ba	1.14 ± 0.06BCab	2.17 ± 0.24Babc	0.31 ± 0.03Bab
	D/D	0.53 ± 0.02Ba	1.34 ± 0.09Ba	2.54 ± 0.17Ba	0.26 ± 0.02Bb
	D/R	0.54 ± 0.03ABa	1.29 ± 0.08Bab	2.42 ± 0.13Bab	0.27 ± 0.01Bb
	D/S	0.52 ± 0.02Ba	1.30 ± 0.04Bab	2.50 ± 0.09Bab	0.26 ± 0.01Bb
	*P: F_*W*_*	**0.000**	**0.000**	**0.000**	**0.000**
	*P: F_*N*_*	**0.000**	**0.000**	**0.000**	**0.000**
	*P: F_*C*_*	0.788	**0.003**	**0.045**	**0.037**
	*P: F_*W*_*_ × **N**_	0.854	**0.000**	**0.000**	**0.000**
	*P: F_*W*_*_ × **C**_	0.129	0.159	0.681	0.534
	*P: F_*N*_*_ × **C**_	**0.031**	**0.008**	0.084	**0.025**
	*P: F_*W*_*_ × **N** × **C**_	**0.026**	0.998	0.460	0.060

*Values are means ± SE (n = 5). L/S, the ratio of leaf to stem dry matters; L/R, the ratio of leaf to root dry matters; S/R, the ratio of stem to root dry matters; B/A, the ratio of belowground to aboveground dry matters; 100% FC, 100% field capacity; 30% FC, 30% field capacity; DD, DR, and DS indicated that D. odorifera planted with D. odorifera, D. regia, and S. mahagoni under the root system interaction, respectively; D/D, D/R, and D/S indicated D. odorifera planted with D. odorifera, D. regia, and S. mahagoni under the root system isolation, respectively; F_W_, water effect; F_N_, nitrogen effect; F_C_, species interaction effect; F_W × N_, the interaction effect of water and nitrogen; F_W × C_, the interaction effect of water and species interaction; F_N × C_, the interaction effect of nitrogen and species interaction; F_W × N × C_, the interaction effect of water, nitrogen, and species interaction. Line mixed model was conducted to evaluate the influence of different factors and their interaction effects. Values followed by different lowercase in the same column are significantly different at P < 0.05 among different species interaction models under the same water and N application; Values followed by different uppercase in the same column are significantly different at P < 0.05 among different water and N application under the same species interaction model. Significant P values are in bold*.

**Table 2:** Obtained effects of *D. odorifera* in regard of percent increase or decrease (%) due to drought or nitrogen application when exposed to different species interaction models.

**Water and *N* level**	**Interaction models**	**H (%)**	**Branch (%)**	**LA (%)**	**LDM (%)**	**SDM (%)**	**RDM (%)**	**TDM (%)**	**L/R (%)**	**S/R (%)**	**B/A (%)**	**WUEi (%)**	**CAT (%)**	**SOD (%)**	**POD (%)**	**Proline (%)**	**NSC (%)**	**Soluble sugar (%)**	**Starch (%)**
30 VS. 100% FC	DD	−60.44	−41.59	−29.82	−69.58	−72.49	−54.42	−67.67	−34.35	−40.35	64.29	−9.64	43.41	46.52	42.52	202.22	19.29	82.96	−42.78
	DR	−51.85	−47.37	−22.19	−59.01	−64.79	−28.52	−55.58	−41.09	−50.00	88.89	77.29	−7.31	23.89	43.01	163.32	25.51	82.92	−30.41
	DS	−59.57	−50.00	−29.28	−53.85	−65.34	−33.23	−55.33	−30.16	−47.5	74.07	77.02	51.86	30.81	72.37	181.47	31.24	83.57	−23.17
100%FC + N VS. 100%FC	DD	32.46	84.96	29.97	31.35	102.26	6.97	61.09	19.85	85.09	−35.71	0.50	−39.84	−29.73	−36.48	−6.02	3.98	62.88	−53.43
	DR	13.84	45.11	23.24	33.40	34.47	−13.18	24.15	72.87	61.38	−37.04	25.55	−32.38	−32.40	−17.97	5.25	3.38	67.08	−58.68
	DS	32.15	66.18	27.09	36.01	88.51	−0.18	55.22	41.27	96.67	−40.74	8.53	−35.18	−5.63	11.84	21.12	17.73	78.29	−45.23
30%FC + N VS. 30%FC	DD	34.81	12.12	21.9	77.49	94.97	48.52	75.82	18.60	30.15	−21.74	39.59	−13.57	−15.11	−17.08	−31.98	−13.06	−15.73	−4.47
	DR	3.45	11.43	5.58	44.18	52.83	−2.18	32.02	43.42	53.66	−33.33	1.77	−3.57	−21.07	8.43	−25.58	−10.69	−8.20	−17.11
	DS	42.73	19.12	32.48	64.82	122.33	25.80	75.24	29.55	72.22	−34.04	42.28	−40.99	−26.17	−26.25	−37.73	−15.65	−10.78	−27.76

*H, height; Branch, the number of branch; LA, leaf area; LDM, leaf dry matter; SDM, stem dry matter; RDM, root dry matter; TDM, total dry matters; L/S, the ratio of leaf to stem dry matter; L/R, the ratio of leaf to root dry matter; S/R, the ratio of stem to root dry matter; B/A, the ratio of belowground to aboveground dry matters; WUEi, water-use efficiency; CAT, catalase activity; SOD, superoxide dismutase; POD, peroxidase; Proline, proline content; NSC, leaf non-structural carbohydrates; Soluble sugar, Soluble sugar content; Starch, starch content. 100% FC, 100% field capacity; 30% FC, 30% field capacity; 100% FC + N, 100% field capacity and N application treatment; 30% FC + N, 30% field capacity and N application treatment. DD, DR, and DS indicated that D. odorifera planted with D. odorifera, D. regia, and S. mahagoni under the root system interaction, respectively*.

Under the root system isolated models (i.e., D/D, D/R, and D/S), insignificant differences in H, LA, and the number of branches and leaves (Figure 3), LDM, SDM, and TDM (Figure 4) among all interaction models were found in all treatments (i.e., 100% FC, 30% FC, 100% FC + N, and 30% FC + N). Under the 100% and 30% FC conditions, the H, SDM, LDM, and TDM of *D. odorifera* seedlings under the D/D models were significantly higher than those under the DD models. In contrast, these parameters showed lower values under D/R and D/S models than under DR and DS models. Under the 100% FC + N condition, H, LA, LDM, SDM, and TDM of *D. odorifera* under the D/S model were lower than that of seedlings under the DS model, whereas *D. odorifera* under the D/R model had higher values than that under the DR model (Figures 3A,E, 4A,B,D, Table 1). Under the 30% FC + N condition, the H, LA, LDM, SDM, and TDM of *D. odorifera* under the D/D model were higher than that under the DD model, whereas these parameters of *D. odorifera* under the D/S model were lower than that under the DS model. The root system interaction planting models collectively had more positive effects on the growth and development of *D. odorifera* than the root system isolated models when *D. odorifera* was planted with different niche neighbors.

### Effects of Water, N Application, and Species Interaction on Gas Exchange and Photosynthetic Pigment Contents

**Figure 6:**
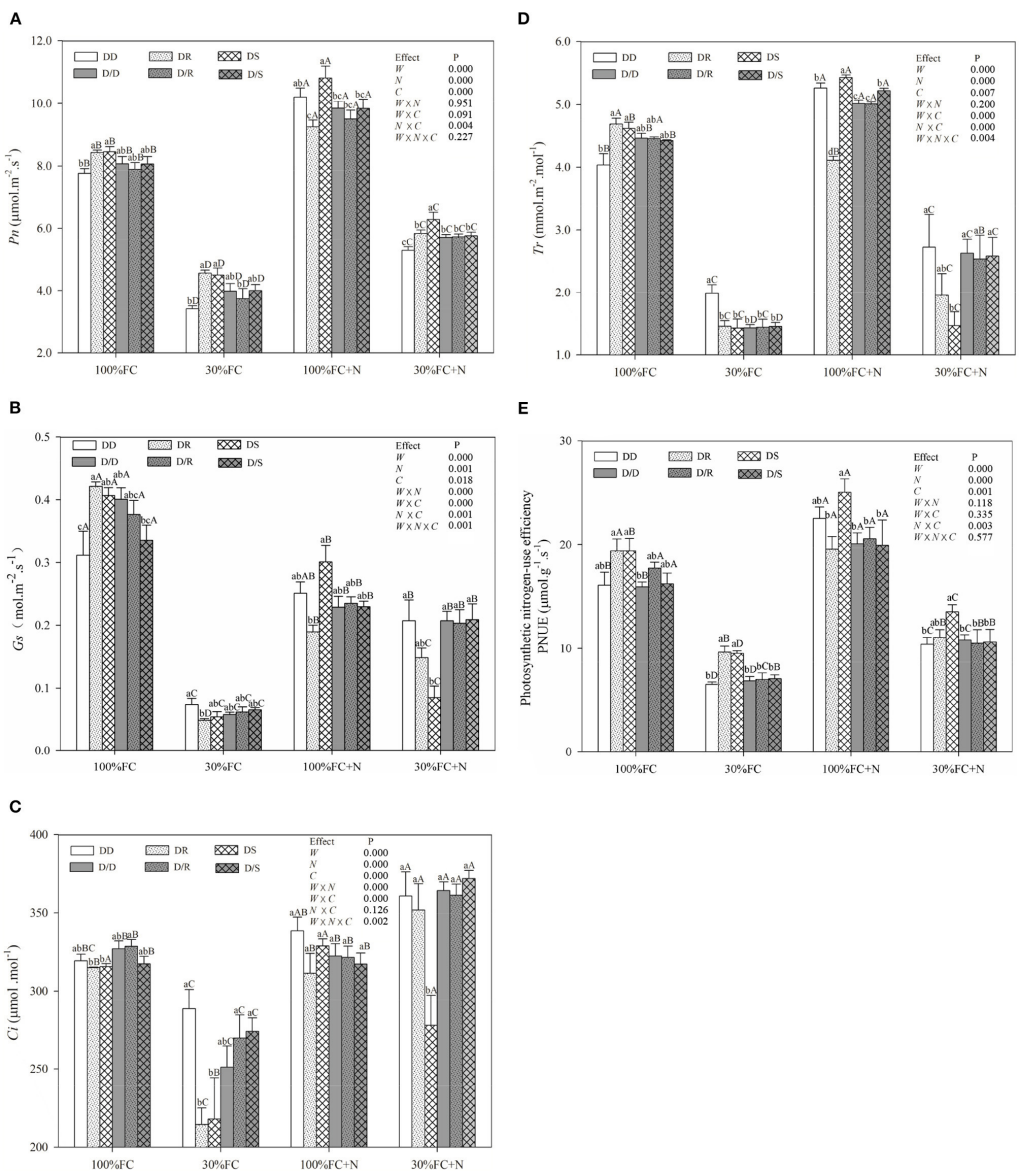
Effects of water, N application, and species interaction on net photosynthetic rates [*Pn*, **(A)**], stomatal conductance [*Gs*, **(B)**], intercellular CO_2_ concentration [*Ci*, **(C)**], transpiration rate [*Tr*, **(D)**], and photosynthetic nitrogen-use efficiency [PNUE, **(E)**] of *D. odorifera*. Treatments of water, N application, species interaction, data description, and statistics are shown in Figure 3.

**Figure 7:**
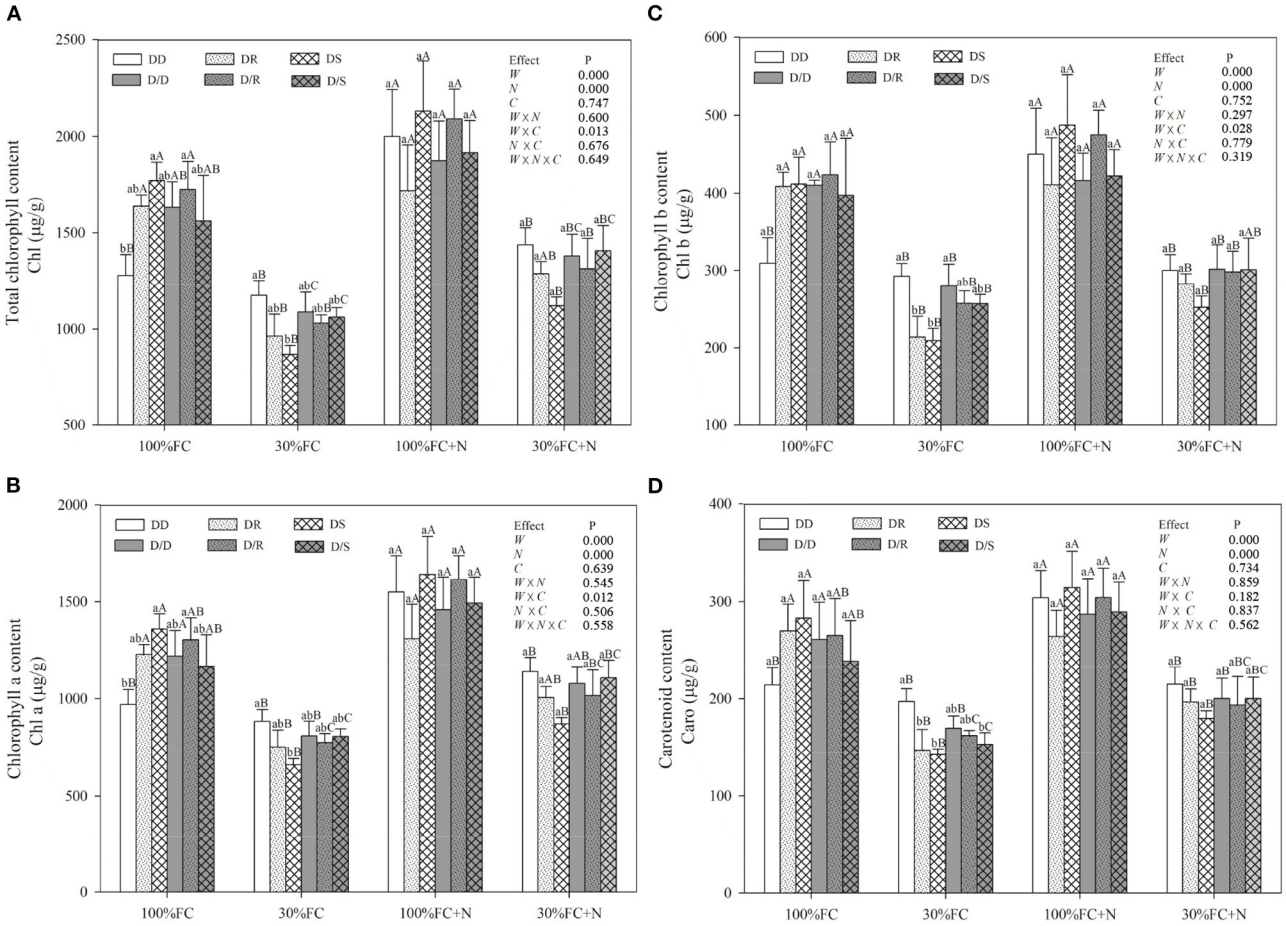
Effects of water, N application, and species interaction on total chlorophyll content **(A)**, chlorophyll a content **(B)**, chlorophyll b content **(C)**, and carotenoid content **(D)** of *D. odorifera*. Treatments of water, N application, species interaction, data description, and statistics are shown as in Figure 3.

Under the root system interaction models, *Pn, Tr*, PNUE, and photosynthetic pigment contents of *D. odorifera* seedlings under the DD model were slightly or notably lower than that of seedlings under the DR and DS models under the 100% FC condition (Figures 6, 7). Meanwhile, these parameters of *D. odorifera* seedlings under the DD model also showed lower values than those of seedlings under the D/D model. Compared with 100% FC condition, drought stress decreased *Pn, Tr*, PNUE, and photosynthetic pigment contents of *D. odorifera* in all interaction models. *Pn* under the DD, DR, and DS model was significantly decreased by 55.79, 45.86, and 46.93%, and PNUE decreased by 59.60, 50.25, and 50.96%, respectively. In addition, *Pn* and PNUE of *D. odorifera* had a higher value under the DR and DS models than under the DD model in 30% FC condition. N application increased *Pn* and PNUE of *D. odorifera* in all interaction models under the 100% and 30% FC conditions (Figure 6A). Under the 100% FC + N condition, *Pn* under the DD, DR, and DS model was significantly increased by 31.66, 9.92, and 27.02%. PNUE increased by 40.01, 0.84, and 31.42% compared with 100% FC condition, respectively. *D. odorifera* under the DR and DS models had lower and higher *Pn*, PNUE, and photosynthetic pigment contents than those under the D/R and D/S models (Figures 6A, 7), respectively. Under the 30% FC + N condition, *Pn* and PNUE of *D. odorifera* increased as the DD, DR, and DS models. However, insignificant differences in gas exchange and photosynthetic pigment contents were observed among the interaction models under the root system isolated planting models (Figures 6, 7). Water, N application, species interaction, and their interaction significantly affected gas exchange parameters of *D. odorifera*.

### Effects of Water, N Application, and Species Interaction on WUEi, Leaf NSC, RWC, Antioxidant Enzymes Activities, and Proline Content

**Figure 8:**
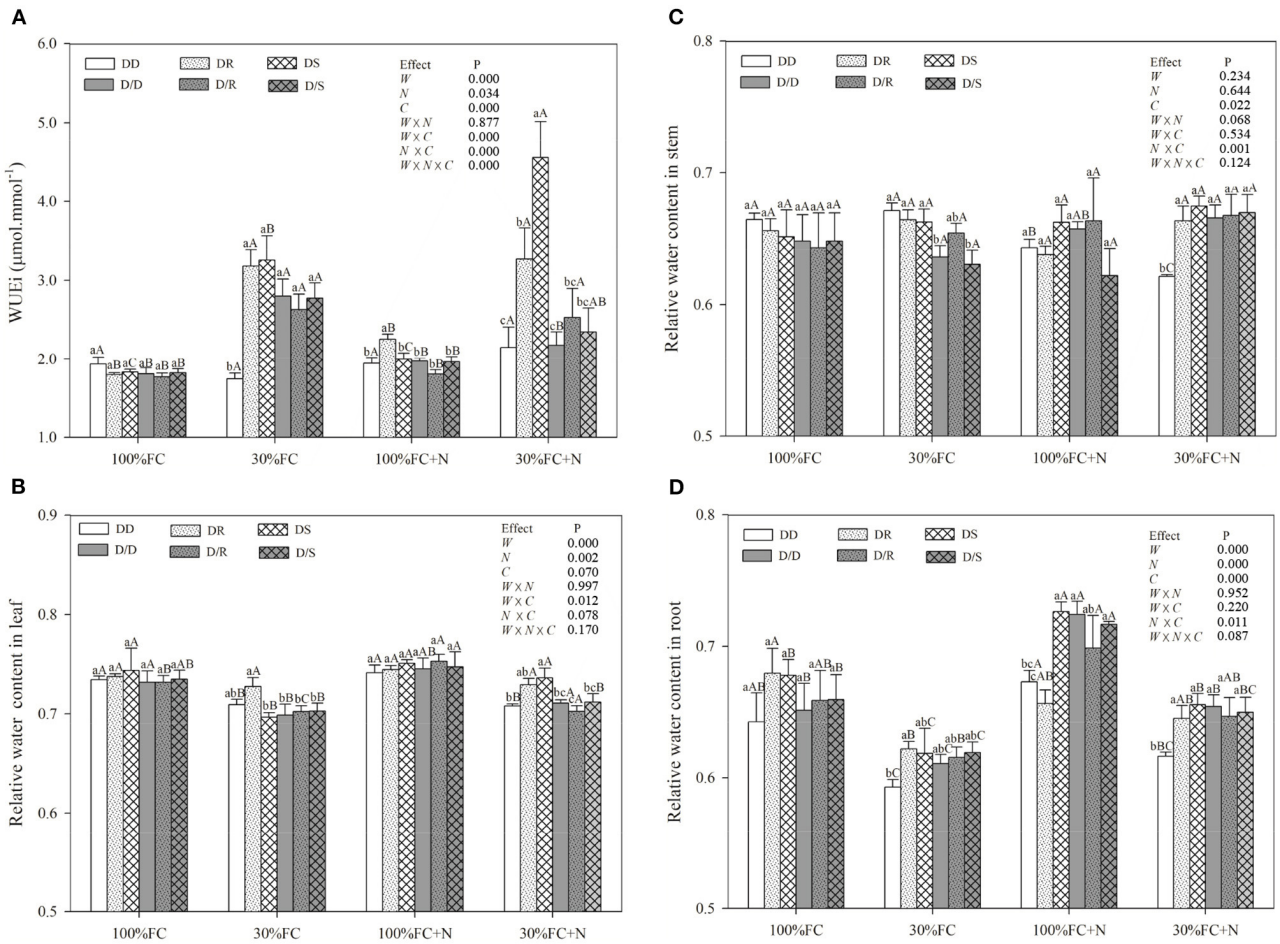
Effects of water, N application, and species interaction on leaf water-use efficiency (WUEi) **(A)**, leaf relative water content **(B)**, stem relative water content **(C)**, and root-relative water content **(D)** of *D. odorifera*. Treatments of water, N application and species interaction, data description, and statistics are shown as in Figure 3.

**Figure 9:**
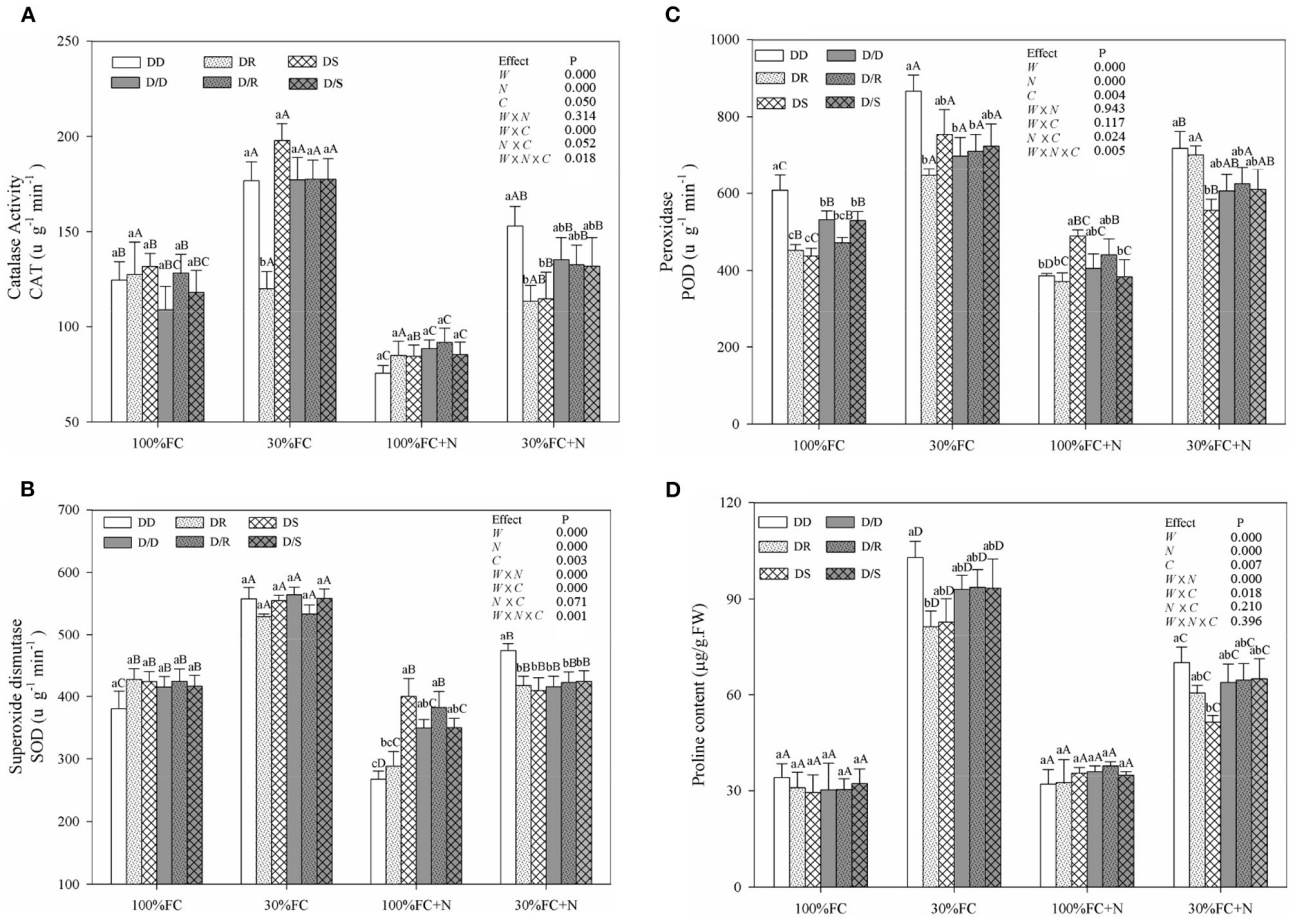
Effects of water, N application, and species interaction on catalase activity (CAT) **(A)**, superoxide dismutase (SOD) **(B)**, peroxidase (POD) **(C)**, and proline content **(D)** of *D. odorifera*. Treatments of water, N application and species interaction, data description, and statistics are shown as in Figure 3.

**Figure 10:**
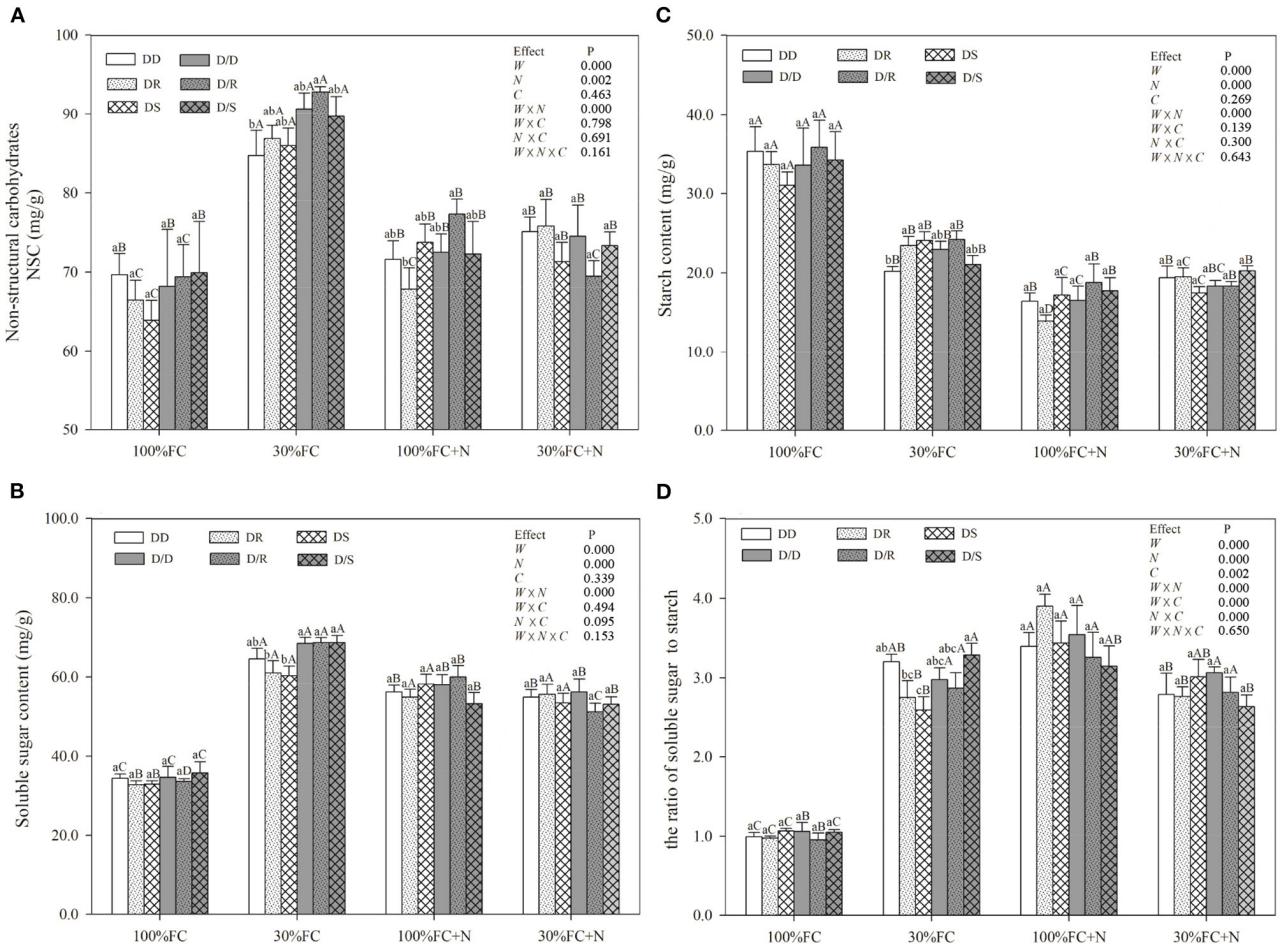
Effects of water, N application, and species interaction on leaf non-structural carbohydrates **(A)**, soluble sugar content **(B)**, starch content **(C)**, and the ratio of soluble sugar content to starch content **(D)** of *D. odorifera*. Treatments of water, N application, species interaction, data description, and statistics are shown as in Figure 3.

Drought stress promoted slightly or notably WUEi under the DR and DS models and increased CAT, SOD, POD, and proline content under all models (Figures 8A, 9). The greater increases in SOD activity and proline content but fewer increases in NSC and WUEi in drought-stressed *D. odorifera* seedlings under the DD model were found compared with those seedlings under DR and DS models (Table 2). *D. odorifera* under the DD model possessed lower WUEi, leaf NSC, and starch content but higher activities of SOD and POD, proline content, soluble sugar content, and the ratio of soluble sugar to starch than those seedlings under the DR and DS models in the 30% FC condition (Figures 9B–D, 10). N application in the 100% FC condition increased NSC of *D. odorifera* leaves under the DS model (Figure 10A). In addition, *D. odorifera* under the DR model had the lowest NSC among all interaction models. N application under the 30% FC condition significantly increased WUEi of *D. odorifera* under root system interaction models but decreased CAT, POD, and proline content. In addition, a less increase in WUEi and fewer decreases in activities of CAT and POD and proline content of *D. odorifera* were observed in the DR model compared with the DD and DS model (Table 2). WUEi of *D. odorifera* increased significantly, and SOD, POD, and proline content decreased as the DD, DR, and DS models (Figures 8A, 9). However, the RWC (Figures 8B–D), WUEi, activities of CAT, SOD, and POD, proline content, and leaf NSC of *D. odorifera* among the D/D, D/R, and D/S models, similar to the abovementioned physiological indicators, showed insignificant differences (Figures 8–10).

### Comprehensive Evaluation of *D. odorifera* Response to Different Species Interaction

As shown in Table 3, under the 100% and 30% FC conditions, the *E* values of *D. odorifera* under the DR and DS models were significantly higher than that of seedlings under the DD model (the *E* values of *D. odorifera* ranking were as follows: DD < DR, DS). In addition, few differences were found in the *E*-values of *D. odorifera* between the DR and DS models under the 100% FC condition. However, the *E*-values of *D. odorifera* under the DS model were significantly higher than that of other interaction models under the 100% FC + N condition (the *E*-values of *D. odorifera* ranking were as follows: DD, DR < DS). Under the 30% FC + N condition, the *E*-value of *D. odorifera* increased gradually with the DD, DR, and DS models (the *E*-values of *D. odorifera* ranking were as follows: DD < DR < DS).

**Table 3:** The value of the comprehensive index [C(μ)], subordinate function value X(μ), and comprehensive evaluation value (*E*) for *D. odorifera* exposed to different species interaction models under various water and *N* application conditions.

**Water and fertilizer level**	**Species interaction**	**C (*1*)**	**C (*2*)**	**X (*1*)**	**X (*2*)**	***E***
100% FC	DD	−5.87	−0.19	0.000	0.430	0.057
	DR	2.53	2.08	0.912	1.000	0.924
	DS	3.34	−1.9	1.000	0.000	0.867
30% FC	DD	−14.75	0.03	0.000	0.526	0.068
	DR	7.76	0.75	1.000	1.000	1.000
	DS	6.99	−0.77	0.966	0.000	0.841
100% FC + N	DD	−0.05	−2.89	0.492	0.000	0.380
	DR	−4.79	1.2	0.000	0.893	0.203
	DS	4.84	1.69	1.000	1.000	1.000
30% FC + N	DD	−4.85	−1.31	0.000	0.000	0.000
	DR	−0.47	2.31	0.431	1.000	0.507
	DS	5.32	−0.99	1.000	0.088	0.878

*Treatments of water, N application, and species interaction are shown as in Table 1*.

## Discussion

### Responses of *D. odorifera* to Different Niche Neighbors Under the Root Interaction Models

The growth and phenotype of plants would be optimized with their social status and neighborhood variation because of resource availability (Duan et al., 2014; Abakumova et al., 2016). The present study revealed that different niche neighbors altered the morphological plasticity of *D. odorifera* under root system interaction models. *D. odorifera* showed better performances in morphology (e.g., H, number of branch and leaves, biomass accumulation, and B/A and RCI > 0) in the inter-specific interaction models (DR and DS) compared with those in the intra-specific interaction models (DD) under the 100% FC and 30% FC conditions (Figures 3–5, Table 1). These findings indicated that the growth of *D. odorifera* markedly benefited from *D. regia* and *S. mahagoni*, whereas they were inhibited by neighboring *D. odorifera*. It confirmed Hamilton's kin selection theory, which indicated that plants might decrease growth (e.g., reduce input to root) when roots experienced genetically similar or equivalent neighbors (Hamilton, 1964). Previous studies have shown that increased B/A could enhance competitiveness for belowground resources (Bennett et al., 2012). The high biomass allocation in the root system of *D. odorifera* under the inter-specific interaction models might optimize the ability to absorb resources by increasing biomass allocation of the root system in response to different niche neighbors under drought conditions. Different niche neighbors would optimize the ability of *D. odorifera* to absorb resources by increasing root biomass allocation, especially under drought conditions, and thus improved the competitiveness of *D. odorifera*. On the other hand, N-fertilized *D. odorifera* showed the best growth performance (e.g., higher H, branch number, leaf number, LA, and B/A and RCI > 0) under the DS model among all interaction models under the well-watered condition. This finding implied that the presence of non-Leguminous Family *S. mahagoni* promoted the growth and development of Leguminous Family *D. odorifera*. In addition, a previous study suggested that greater niche differentiation would reduce niche overlap and improve resource utilization. Therefore, neighboring *D. regia* and *S. mahagoni* of *D. odorifera* were more beneficial to its growth than neighboring *D. odorifera* due to niche complementarity, particularly in restricted and harsh environments (del Río et al., 2014; Zuppinger-Dingley et al., 2014; Cattaneo et al., 2018). Similarly, the growth traits of *D. odorifera* gradually increased slightly or significantly with the DD, DR, and DS models under the 30% FC + N condition, which indicated that distant-stranger neighbors were favorable to the growth of *D. odorifera* in comparison to the close-relative neighbors. Thus, the performance of *D. odorifera* would depend on the degree of niche complementarity among interactive species (Pezzola et al., 2019). Collectively, our results suggested that the growth of *D. odorifera* would respond differentially to different niche neighbors, and PCA confirmed that *D. odorifera* between intra-specific interaction and inter-specific interaction models could be separated from each other.

Previous studies have demonstrated that neighbors could affect the physiological performances and N-use efficiency of the target species, including the chlorophyll (Guo et al., 2018), photosynthetic parameters (Goisser et al., 2016), and PNUE (Duan et al., 2014; Yu et al., 2017). Similarly, the inter-specific interaction between *Populus purdomii* and *Salix rehderiana* improved the photosynthetic capacity of *S. rehderiana* than the intra-specific interaction, indicating that *S. rehderiana* benefited from the presence of *P. purdomii*, particularly under the N-poor condition (Song et al., 2017). Therefore, our studies demonstrated that photosynthetic capacity and N-use efficiency of *D. odorifera* were negatively limited by *D. odorifera* (intra-specific interaction) but positively promoted by the *D. regia* and *S. mahagoni* (inter-specific interaction) under no-N application conditions (Figure 4). On the other hand, the height and biomass accumulation could be viewed as indicators of competitiveness, and PCA showed positive correlations between *Pn* and PNUE and these indexes (Figure 2). The highest *Pn* and PNUE under the DS model suggested that the neighboring *S. mahagoni* improved the competitiveness of *D. odorifera*. Furthermore, the *Pn* and PNUE of *D. odorifera* seedlings showed remarkable photosynthetic performances and N-use efficiency with the enlarged degree of niche differentiation among neighbors under the 30% FC + N condition. The results indicated that *D. odorifera* modulated traits in photosynthetic capacity according to its neighbors, and the responses of *D. odorifera* in photosynthetic capacity were associated with its neighbors and their niche differentiation (Burns and Strauss, 2011).

NSC might be an incentive for increased competitiveness (Duan et al., 2014). The increments of foliar soluble sugars, antioxidant enzymes, and proline content had contributed to maintaining normal cellular turgor and osmoregulation when plants encountered stronger competition for water resources (Zrenner and Stitt, 1991; Zhang et al., 2019; He et al., 2020). Furthermore, plants with higher water-use efficiency had greater advantages in excluding neighbors (Craine et al., 2013). In this study, PCA indicated positive correlations between WUEi and leaf NSC (Figure 2). *D. odorifera* exhibited higher WUEi, starch, total NSC, and lower activities of SOD and POD, proline content, soluble sugar, and the ratio of soluble sugar to starch under both DR and DS models than the DD model under the 30% FC condition. This finding suggested that the neighboring *D. regia* and *S. mahagoni* alleviated negative effects caused by drought stress on *D. odorifera*. Therefore, the notable improvement in water utilization of *D. odorifera* under both DR and DS models could help carbon storage (Figures 8A, 9A,B). However, neighboring *D. odorifera* aggravated the intensity of drought stress, then increased ROS, antioxidant enzymes, proline content, and soluble sugar in response to reduce the oxidative damages, and finally blocked the synthesis of NSC starch in *D. odorifera* under the DD model. The results suggested that plants could display different adaptive responses in leaf NSC, antioxidant enzymes, and proline content according to neighbors (Yu et al., 2019).

Taken together, growth and development, physiological, and morphological traits of *D. odorifera* could differentially respond to its neighbors with distinct niche under root system interaction models, particularly under the drought-stressed conditions, which was in agreement with our hypothesis.

### Negligible Responses of *D. odorifera* to Different Niche Neighbors Under the Root Isolated Models

Plants altered related traits to improve their competitiveness in response to aboveground and belowground competition (Murphy and Dudley, 2007). Compared with the root system isolated models under the 100% and 30% FC conditions, *D. odorifera* from inter-specific interaction models under the root system interaction had better performances in growth, biomass accumulation, RCI, *Pn*, PNUE, POD activity, and proline content. This finding indicated that *D. odorifera* under the root system interaction models had more positive effects from neighbors than those under the root system isolated models. The few or insignificant differences in the growth, morphology, and physiology of *D. odorifera* among the D/D, D/R, and D/S models in majority treatments indicated that the responses of *D. odorifera* to different niche neighbors were dominated by belowground interaction rather than the negligible aboveground interaction. However, studies on the interaction in tropical forests indicated that aboveground competition for light and space exceeded that from the belowground competition for nutrients and water because of the limitation of photosynthetically active radiation (Lewis and Tanner, 2000). The light limitation would occur on the blade, wherein the light available for photosynthesis could not meet the photosynthetic capacity of leaves, which was necessary for plant light competition (Craine et al., 2013). Therefore, based on the negligible responses of *D. odorifera* from the aboveground interaction in our experiment, we supposed that light was not fully saturated to activate the aboveground competition mechanism for light.

### Responses of *D. odorifera* to Different Niche Neighbors Were Modified by Drought and N Application Under the Root Interaction Models

Abiotic factors played a key role in the interaction as the primary driving force (Duan et al., 2014; Yu et al., 2019). In the present study, drought-stressed *D. odorifera* seedlings under the DD model showed greater decreases in growth (e.g., H, LA, LDM, SDM, RDM, and TDM) and photosynthetic capacity (*Pn* and PNUE), greater increases in SOD activity and proline content, and fewer increases in NSC and WUEi. These results demonstrated that drought would aggravate the inhibitory effects on *D. odorifera* under the DD model. Similar results have been found in other studies, such that inhibited effects from conspecifics became stronger under drought stress (Chen et al., 2009; Wright et al., 2015; Metz et al., 2016). For example, in one study, the greater inhibition (lower growth and physiological parameters) of *P. cathayana* females was found from intrasexual competition than the intersexual competition when facing variation from well-watered to drought treatment (Chen et al., 2014). These results can be explained by the greater increase in competitive intensity for water that resulted from the similarity of resource utilization, which aided in the capture of a small amount proportion of available water resources (Chen et al., 2014; Calama et al., 2019). Interestingly, the growth and development of *D. odorifera* showed similar performances between the DR and DS models in the 100% FC condition. However, the trend changed by N application, as N fertilized *D. odorifera* under the DS model gained greater positive effects than that under the DR model, and *D. odorifera* under the DR model suffered stronger inhibitory effects (e.g., the value of RCI dropped from positive to negative) under the 100% FC + N condition (Figure 5; Table 2). A related study reported that nutrient supply could increase the competition intensity between *Eucalyptus* and *Acacia mangium* (Bordron et al., 2021). No positive interaction of N-fixing *Alnus rubra* on the growth of *Pseudotsuga menziesii* could be converted to a strong facilitating effect by high soil N availability (Binkley et al., 2003). These are consistent with the stress-gradient hypothesis that interactions with neighbors could be converted from positive to negative in favorable conditions (N application) (Bertness and Callaway, 1994). Therefore, N application could stimulate the competitiveness of Leguminosae Family *D. odorifera* from non-Leguminosae Family *S. mahagoni* and enhance the competitive intensity of *D. odorifera* from Leguminosae Family *D. regia* under a well-watered condition. However, the N application promoted the performances of *D. odorifera* under the DD and DS models and had little effect on *D. odorifera* under the DR model under drought-stressed conditions. These results are consistent with some previous studies, in which negative effects of competition in low resource environments are converted to the positive effects of facilitation by adding resources (e.g., N application; Forrester et al., 2013; Svanfeldt et al., 2017). For example, growth and complementarity effects between mixed-species stands of *Abies alba* and *Picea abies* improved as growing conditions improved (Forrester et al., 2013). Therefore, N application reduced the inhibitory effect of drought stress on *D. odorifera* under the DD and DS models (Tilman, 1987), and N played a key role in the responses of *D. odorifera* under the root system interaction models (Yu et al., 2017; Guo et al., 2018). Song et al. (2017) considered that N altered the relationships between *S. rehderiana* and *P. purdomii* in a glacier retreat area. Therefore, water level and N application could alter *D. odorifera* to different niche neighbors under the root system interaction models, which was also consistent with our hypothesis.

## Conclusions

In conclusion, Leguminosae Family *D. odorifera* can differentially respond to its different niche neighbors under the root system interaction models, and it can benefit from niche differentiation. It should be encouraged to select different niche mixture species during the construction of the *D. odorifera* forest. The water regime and N application may modulate the interactive effects between neighbors. Appropriate N application maybe alleviate the inhibitory effect of drought stress on *D. odorifera* in its mixed forests. Mixture with *S*. *mahagoni* or *D. regia* under both the well-watered and drought conditions with root system interaction is the optimal planting model. However, under the condition of N application, a mixture with *S*. *mahagoni* could be the only optimal planting model.

## Data Availability Statement

The original contributions presented in the study are included in the article/Supplementary Material, further inquiries can be directed to the corresponding author/s.

## Author Contributions

L-SX performed the experiment and wrote the draft manuscript. L-FM assisted in carrying out the experiment in the greenhouse. FY designed the experiment, provided funding, and revised the manuscript. All authors were engaged in this present work.

## Conflict of Interest

The authors declare that the research was conducted in the absence of any commercial or financial relationships that could be construed as a potential conflict of interest.

## Abbreviations

Branch: the number of branch
B/A: the ratio of belowground dry matter to aboveground dry matters
Caro: carotenoid content
CAT: catalase activity
Chl: total chlorophyll content
Chl a: chlorophyll a content
Chl b: chlorophyll b content
*Ci*: intercellular CO_2_ concentration
D: stem diameter
*E*: comprehensive evaluation
DD: interaction models between *Dalbergia odorifera* and *D. odorifera*
D/D: interaction models with partition pots between *D. odorifera* and *D. odorifera*
DR: interaction models between *D. odorifera* and *Delonix regia*
D/R: interaction models with partition pots between *D. odorifera* and *D. regia*
DS: interaction models between *D. odorifera* and *Swietenia macrophylla*
D/S: interaction models with partition pots between *D. odorifera* and *S. macrophylla*
FC: field capacity
*Gs*: stomatal conductance
H: height
LA: leaf area
LDM: leaf dry matter
L/S: the ratio of leaf to shoot dry matter
L/R: the ratio of leaf to root dry matter
N: nitrogen
NSC: non-structural carbohydrates
Number (L): the number of leaves
*Pn*: net photosynthesis rate
PNUE: photosynthetic nitrogen-use efficiency
POD: peroxidase
Proline: proline content
RCI: relative competition intensity
RDM: root dry matter
*Tr*: transpiration rate
RWC: relative water content
RWC(L): leaf relative water content
RWC(R): root relative water content
RWC(S): stem relative water content
SDM: stem dry matter
SLW: specific leaf weight
S/R: ratio of shoot to root dry matter
SOD: superoxide dismutase
Soluble sugar/starch: the ratio of soluble sugar to starch content
TDM: total dry matters
WUEi: intrinsic water-use efficiency.
