# Supplementary material for: Drought and Nitrogen Application Modulate the Morphological and Physiological Responses of Dalbergia odorifera to Different Niche Neighbors
Source: Front Plant Sci. 2021 Jul 2;12:664122. doi: 10.3389/fpls.2021.664122 (PMC8283204; doi:10.3389/fpls.2021.664122)
Supplement: Supplementary file 1 [file Data_Sheet_1.docx]

Supplementary Material

# Supplementary Figure legend

# Figure 1 Effects of water, N application, and species interaction on the growth of *D. odorifera*.

100% FC, 100% field capacity; 30% FC, 30% field capacity; 100% FC + N, 100% field capacity and N application treatment; 30% FC + N, 30% field capacity and N application treatment; DD, DR, and DS indicated that *D. odorifera* planted with *D. odorifera*, *D. regia*, and *S. mahagoni* under the root system interaction, respectively; D/D, D/R, and D/S indicated that *D. odorifera* planted with *D. odorifera*, *D. regia*, and *S. mahagoni* under the root system isolation, respectively.

## Supplementary Figures


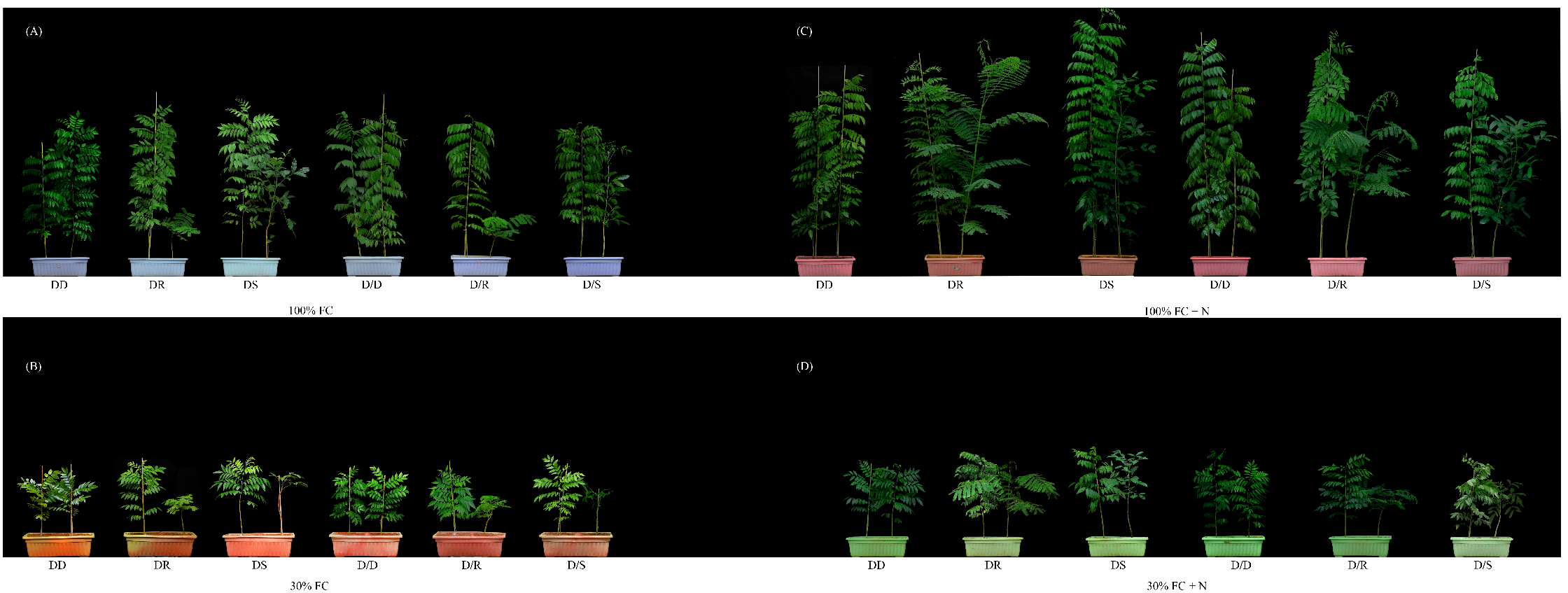
**Figure 1**
